# Supplementary material for: Graphene Oxide-Induced Protein Conformational Change in Nasopharyngeal Carcinoma Cells: A Joint Research on Cytotoxicity and Photon Therapy
Source: Materials (Basel). 2021 Mar 13;14(6):1396. doi: 10.3390/ma14061396 (PMC8001416; doi:10.3390/ma14061396)
Supplement: Supplementary file 1 [file materials-14-01396-s001.pdf]

materials

Supplementary Materials

Graphene Oxide-Induced Protein Conformational Change in Nasopharyngeal Carcinoma Cells: A Joint Research on Cytotoxicity and Photon Therapy

Selvaraj Rajesh Kumar [1], Ya-Hui Hsu [2], Truong Thi Tuong Vi [1], Jong-Hwei Su Pang [2,3], Yao-Chang Lee [4], Chia-Hsun Hsieh [5,6,7,*] and Shingjiang Jessie Lue [1,8,9,10,*]

[1] Department of Chemical and Materials Engineering, Chang Gung University, Wenhua 1st Road, Guishan, Taoyuan 333, Taiwan; rajeshkumarnst@gmail.com (S.R.K.); truongthituongvi005@gmail.com (T.T.T.V.)
[2] Graduate Institute of Clinical Medical Sciences, Chang Gung University, Wenhua 1st Road, Guishan, Taoyuan 333, Taiwan; hyh17@cgmh.org.tw (Y.-H.H.); jonghwei@mail.cgu.edu.tw (J.-H.S.P.)
[3] Department of Physical Medicine and Rehabilitation, Chang Gung Memorial Hospital, Dinghu Road, Guishan, Taoyuan 333, Taiwan
[4] National Synchrotron Radiation Research Center, Hsin Ann Road, Hsinchu City 300, Taiwan; yclee@nsrrc.org.tw
[5] Division of Hematology-Oncology, Department of Internal Medicine, New Taipei Municipal TuCheng Hospital, Jincheng Road, New Taipei City 236, Taiwan
[6] Division of Hematology-Oncology, Department of Internal Medicine, Chang Gung Memorial Hospital at Linkou, Fusing Street, Guishan, Taoyuan 333, Taiwan
[7] School of Medicine, Chang Gung University, Wenhua 1st Road, Guishan, Taoyuan 333, Taiwan
[8] Division of Join Reconstruction, Department of Orthopedics, Chang Gung Medical Center at Linkou, Fusing Street, Guishan, Taoyuan 333, Taiwan
[9] Department of Safety, Health and Environment Engineering, Ming Chi University of Technology, Gongzhuan Road, Taishan, New Taipei City 243, Taiwan
[10] Center for Environmental Sustainability and Human Health, Ming Chi University of Technology, Gongzhuan Road, Taishan, New Taipei City 243, Taiwan
* Correspondence: wisdom5000@gmail.com (C.-H.H.); jessie@mail.cgu.edu.tw (S.J.L.). Tel.: +886-3-2118800 ext. 5489 (S.J.L.); Fax: +886-3-2118700 (S.J.L.).

Citation: Kumar, S.R.; Hsu, Y.-H.; Vi, T.T.T.; Pang, J.-H.S.; Lee, Y.-C.; Hsieh, C.-H.; Lue, S.J. Graphene Oxide-Induced Protein Conformational Change in Nasopharyngeal Carcinoma Cells: A Joint Research on Cytotoxicity and Photon Therapy. *Materials* **2021**, *14*, 1396. https://doi.org/10.3390/ma14061396

Academic Editor: Montserrat Colilla

Received: 4 February 2021
Accepted: 10 March 2021
Published: 19 March 2021

Publisher's Note: MDPI stays neutral with regard to jurisdictional claims in published maps and institutional affiliations.

Copyright: © 2021 by the authors. Licensee MDPI, Basel, Switzerland. This article is an open access article distributed under the terms and conditions of the Creative Commons Attribution (CC BY) license (http://creativecommons.org/licenses/by/4.0/).

S1. Preparation of graphene oxide

In detail, 3 g graphite powder (Sigma-Aldrich, St. Louis, Missouri, USA) was mixed in 400 mL sulfuric acid (H_2SO_4, 95-98%, Scharlab S.L., Barcelona, Spain) with continuous mechanical stirring at room temperature. Then, 3 g potassium permanganate ($KMnO_4$, Nihon Shiyaku Industries Ltd., Osaka, Japan) was slowly mixed into the solution. After $KMnO_4$ addition, the green color vanished, and another portion of $KMnO_4$ (2 g) was added. This process was repeated to achieve a total addition of 5 equivalents of $KMnO_4$ by weights. Then, the appropriate quantity of ice cubes was added to the solution for the exfoliation process and kept overnight. The obtained precipitate particles were washed with deionized (DI) water until the solution reached neutral pH. Then, the particles were dried in a vacuum oven at 60 °C to acquire bulk GO nanosheets.

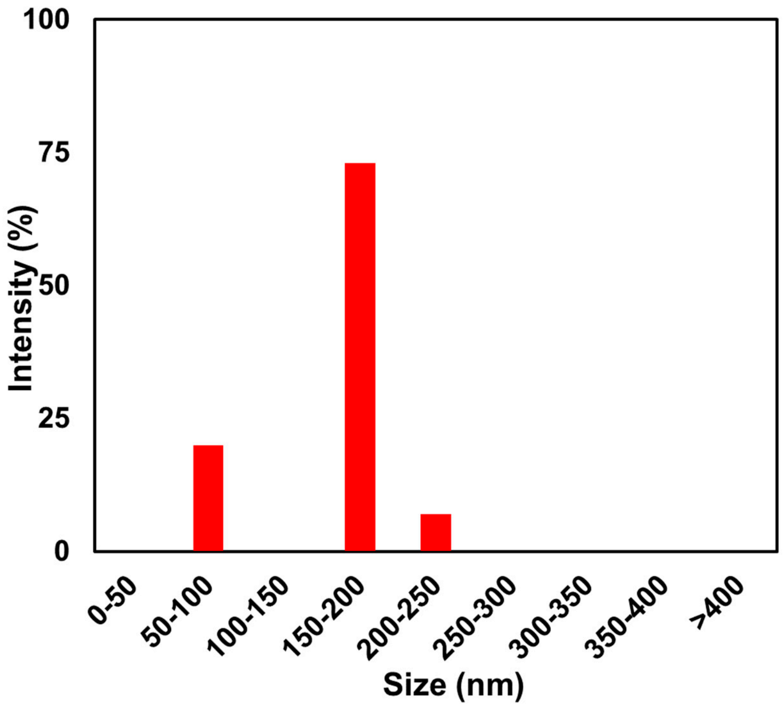

Figure 1. Particle size distribution of nanometric graphene oxide.
